# Does believing in different types of religion affect subjective wellbeing? Analysis of the public data of the Taiwan Social Change Survey

**DOI:** 10.3389/fpsyg.2022.1054566

**Published:** 2022-12-07

**Authors:** Yu Ding, Weidong Huo, Yaning Jin

**Affiliations:** ^1^School of Politics and International Studies, Central China Normal University, Wuhan, China; ^2^Institute of Guangdong, Hong Kong and Macao Development Studies, Sun Yat-sen University, Guangzhou, China; ^3^School of Political Science and Public Administration, Wuhan University, Wuhan, China

**Keywords:** types of religious beliefs, urban and rural areas, mediating model, religious activities, subject wellbeing

## Abstract

Previous studies have revealed the impact of objective material conditions and psychological factors, such as the influence of religion on subjective well-being, but have disregarded the role played by differences of religious types formed in Asian cultural and historical contexts. Against this background, the present study aims to examine the association between religious type and subjective wellbeing and its mechanisms – the mediating role of frequency of religious activity and the moderating role of urban-rural areas. This study used Taiwan Social Change Survey (TSCS) 2018 data for researching. The results show that the discrepancy in the frequency of participation in religious activity caused by different types of religious belief will influence personal wellbeing. Respondents who adhere to institutional religion have a higher frequency of participating in religious activities, which has a positive impact on subjective wellbeing. Moreover, further examination shows that urban–rural areas play an important moderating role: respondents living in urban areas are more inclined to participate in religious activities frequently to gain a sense of wellbeing.

## Introduction

Subjective wellbeing is a general term for people's personal assessments of their own lives, events, bodies, minds, and living environments (Oishi et al., 2021). A large number of sociologists, psychologists, and economists have discussed the importance of wellbeing (Dolan, 2014). Common experience describes that changes in both objective material life and the satisfaction of subjective spiritual needs affect wellbeing. Focusing on subjective psychological factors, the relationship between religion and subjective wellbeing has been an important topic of discussion among researchers. Many studies have meticulously analyzed the intrinsic factors of religion that influence wellbeing and examined the relationship between the variables of religious beliefs (James and Chris, 2013), religiosity (Hoogeveen et al., 2022), and wellbeing. However, existing studies have neglected to consider the multiple types of religions formed through long-term historical and cultural traditions in Asia and have failed to fully reveal the relationship between different types of religions and subjective wellbeing in Asia. Therefore, the goal of this article is to reveal the underlying mechanism by which the type of religiosity affects individuals' subjective wellbeing using a quantitative research method.

## Literature review

Research on the relationship between wellbeing and religion is developing rapidly. “Wellbeing” is often used to refer to actual experience (Gasper, 2010). Conceptually, “wellbeing” emphasizes an individual's experience. How does religious belief, as a particular spiritual experience of the individual, affect subjective wellbeing?

When examining the factors that influence subjective wellbeing, researchers first focused on the correlation between objective material conditions and subjective wellbeing. According to early economics, the enhancement of people's economic level will change their quality of life, and the improvement of basic public services, employment, social security, and other aspects will enable individuals to have higher life satisfaction and wellbeing. Unemployment (Clark and Oswald, 1994), inflation of prices (Di Tella et al., 2001), democracy (Dolan et al., 2008), political freedom (Blanchflower and Oswald, 2004), and living environment (Levinson, 2012) all have an impact on subjective wellbeing, as proven by researchers. The economist Easterlin studied the relationship between income and subjective wellbeing in different countries and found no significant correlation between economic development and subjective wellbeing; the level of wellbeing in poor countries is almost as high as that of rich countries. This discovery is called the “Easterlin paradox” (Easterlin, 1974) and has caused extensive controversy among scholars in economics, sociology, and psychology. It has also made the academic community focus on the impact of subjective experience, value orientation, and other factors on subjective wellbeing.

As the relationship between personal subjective experience and wellbeing has received increasing attention from researchers, the role of individual experiences, emotions, and values on wellbeing has become the focus of research, and the influence of personal health (Deaton, 2008), personality traits (Peterson et al., 2007), and social relations (Helliwell, 2003) on personal subjective wellbeing has been continuously analyzed by researchers. Subjective factors involve personal spiritual needs, among which the influence of religious belief has increasingly become a focus of empirical research (Abu-Raiya and Agbaria, 2016). However, studies on the relationship between religious belief and subjective wellbeing have failed to form a consistent view, and some conclusions are even contradictory. Some scholars insist that religious beliefs provide individuals with psychological sustenance and a certain sense of belonging. People who have religious beliefs are more likely to experience wellbeing in life, and there is a positive effect between them (Abdel-Khalek, 2006). By contrast, some scholars argue that no relationship exists between religious belief and subjective wellbeing (Abdel-Khalek and Naceur, 2007), while other studies suggest that analyzing the relationship between religion and mental health requires distinguishing whether a person believes in religion and it has been found that religious persons who have a symbolic attitude toward religion score higher on positive aspects of mental health (wellbeing) (Bänziger et al., 2005).

Different research conclusions have significance for the development of this study. The contradictory results of the aforementioned studies on the relationship between religious belief and wellbeing are mainly due to differences in the methods of measuring variables, mechanisms, and scenarios. In terms of measurement methods, previous research has used a variety of different measures of religiosity, including religious attitudes, religious experience, religious conversion, and religious behavior. Single- and multi-item scales were also used to measure subjective wellbeing (Lewis and Cruise, 2006). These different measurements of variables have led to the diversity in results. In the analysis of specific mechanisms, endogeneity problems exist with regard to the influence of religious belief on subjective wellbeing. For example, religious behavior is affected by the type of religion and the degree of piety. However, there is no clear distinction regarding whether wellbeing is obtained directly from religious belief or indirectly from religious behavior (such as reciting doctrine or participating in religious charitable acts). In addition, the research results of different research scenarios are discrepant. Existing research has mostly selected countries that support institutional religions. However, due to the diverse folk beliefs in East Asian countries, especially in Chinese society (Laliberte, 2016), the applicability and explanatory power of relevant research findings may be problematic. Therefore, this study performed an empirical analysis based on the aforementioned research situation and discusses the mechanism by which religious belief influences subjective wellbeing.

## The division of religious beliefs and types in Taiwan

Influenced by multicultural traditions, different regions have developed their own distinctive cultures and religions. Many researchers have examined different types of religions and classified them according to different criteria. For example, Max Weber distinguished different religious affiliations, including Calvinism, Pietism, Methodism, and the Baptist sects, and he identified the religious foundations of worldly asceticism (Weber, 2002). However, this classification mainly focuses on large religions with a long history (such as Christianity, Islam, and Confucianism) and emphasizes the different affiliations of large religions and their religious doctrines while relatively ignoring the pluralistic folk beliefs of the Asian region. With the deepening of cross-regional comparative studies on religions, researchers have found that superstition and folk belief have an irreplaceable position in the societies of Asian countries (Reid and Tamaru, 1996). In China, which is influenced by traditional culture and religious policies, five major legal religions recognized by the state have emerged, namely Buddhism, Taoism, Catholicism, Christianity (Protestantism), and Islam. At the same time, a large number of folk beliefs still exist in civil society that is in the gray zone of government management (Yang, 2006). To more accurately classify the types of religions in Asian countries, Ching-Kun Yang argued that Chinese religions were not only universal but also had unique qualities. This uniqueness is reflected in Chinese folk beliefs, which correspond to the officially promoted Confucian ethics and are more acceptable to civil society than the moral allegories of Confucianism. Folk beliefs and their mystery are deeply embedded in the lives of ordinary people (Yang, 1967). Based on the characteristics of Asian religions, the aforementioned studies provide useful reflections for classifying Asian pluralistic religions.

The types and structures of religious beliefs in Taiwan are quite distinctive and have gradually formed two major categories: indigenous folk beliefs and world religions introduced at a later stage. The proportion of people with religious beliefs is high in Taiwan, and almost every county and city have places of worship. Residents believe in world religions, including Buddhism, Islam, and Christianity, as well as indigenous religions, such as Mazu (originally the goddess of the sea and now worshiped as an all-powerful protective deity) and the Royal Lords (Wangye, the deity of plague now invoked to counter all manners of calamities) (Katz, 2003).

From the perspective of the religious development process in Taiwan, pluralistic religious organizations were influenced by religious management policies in different periods, making the development of indigenous religions different from that of the religions later introduced. Beginning in 1980, Taiwan gradually deregulated religious organizations within its jurisdiction, and the number of religious organizations has increased dramatically since ~1990 (Qi, 2019). Theoretically, in a completely free religious market (Stark and Finke, 2000), institutional religions are more mature in the ways and means of preaching and attracting followers, and the development of folk beliefs is more easily squeezed by the expansion of institutional religions. However, because the local government in Taiwan adopted a strategy of “grasping the big and releasing the small” in religious management, institutional religions with strict organization and doctrines were more restricted, while folk beliefs received a more relaxed space for development. In this policy environment, folk religions and guild religions in Taiwan attracted more followers by taking advantage of their innate local advantages (Lu et al., 2008). At the same time, with the external environment of electoral politics of Taiwan and the reliance of the Kuomintang patronage network on nongovernmental organizations, diffused religion had a huge “market share” in Taiwan (Katz, 2003).

The changes in religious management policies and the development of religious organizations in Taiwan have gradually formed the current religious pattern in which multiple religions coexist. The pluralistic religious beliefs make Taiwan a typical case for observing East Asian society and Chinese society. More importantly, the huge folk belief groups provide an important foundation for this work to study the influence of religious beliefs on subjective wellbeing.

## Research hypothesis

### Types of religious belief and religious activity

Experience shows that there are significant differences in religious activities organized by different types of religions. Religious activities refer to the collective activities of religious groups and religious believers' own worship, prayer, and other activities. Participation in religious activities may allow individuals to receive social assistance from religious groups and make religious believers feel psychologically close to the object of their faith.

The current classification of religious types is mainly based on the basic philosophical concepts and theological categories of religions, including Buddhism, Christianity, and Islam (Liu, 2008). However, this classification often makes it difficult to find the corresponding coordinates for the widespread folk beliefs in East Asia. Especially, under the influence of diverse histories and cultures, different countries have formed their own distinctive indigenous beliefs. In Asian countries, including China, India, and Japan, people have multiple folk beliefs in addition to the three major religions of the world, namely, Buddhism, Islam, and Christianity. By combing through the literature on Chinese religions, some researchers have noted the pluralistic religions formed in China's special historical and cultural context. After in-depth observation and research of different religions in China, Ching-Kun Yang categorized the diverse religions in Chinese society according to their religious beliefs, ritual symbols, and organizational characteristics and divided them into institutional religion and diffused religion. Institutional religions include Islam, Christianity, and Catholicism. They have their own relatively independent systems, rituals, and organizations and hold religious activities regularly. Some of them even have certain binding regulations on the behavior of believers. The other category is diffused religion. With its theology, rituals and organization intimately merged with the concepts and structures of secular institutions and other aspects of the social order (Yang, 1967), diffused religion mainly refers to various folk beliefs and ancestor worship.

In addition to the disparities in the characteristics of the two types of religious beliefs, they differ in their religious activities and rituals. Compared with institutional religion, which has more frequent, stable, and standardized ritual activities, the activities and rituals of diffused religion are usually not fixed. Due to the distinction in the organizational form and frequency of religious activities between different types of religions, believers also have distinctive features of participation in religious activities, which create diversity in the degree of religious commitment of believers and ultimately affect changes in their wellbeing. Believing in different types of religions may contribute to discrepancies in believers' enthusiasm for participating in religious activities. Therefore, participation in religious activities may play a mediating role between types of religious belief and subjective wellbeing. When examining the relationship between religious types and subjective wellbeing, it is necessary to include the variable of religious activities and pay attention to whether and how it affects wellbeing.

This study proposes three research hypotheses, which are as follows:

**Hypothesis 1:** Compared with diffused religion, institutional religion can significantly increase the frequency of participation in religious activity.
**Hypothesis 2:** Compared with diffused religion, institutional religion can significantly improve subjective wellbeing.
**Hypothesis 3:** The frequency of participation in religious activity has a mediating effect on the relationship between types of religious belief and subjective wellbeing.

### Urban and rural areas, religious beliefs, and subjective wellbeing

Religious beliefs, religious practices, and subjective wellbeing are influenced by the context in which one lives, with differences between urban and rural areas at the regional and national levels creating the most systematic differences in living environments. However, the relationship between urban–rural areas and subjective wellbeing is still controversial. Some studies have suggested that increasing urbanization will reduce residents' wellbeing (Hudson, 2006). Urban residents generally face multiple pressures, such as housing and traffic. Consequently, the larger the city is, the lower the wellbeing of its residents (Knight, 2009). Other studies have argued that compared with rural areas, urban areas have more abundant education, medical, and other resources, and their economic income level is higher. This material basis guarantee greatly improves residents' wellbeing (Easterlin and Angelescu, 2011). In addition, interesting research has found that the satisfaction of urban residents' social status and spiritual needs has a significant positive impact on subjective wellbeing after all types of basic material security are met (Wei, 2012).

Overall, there are certain gaps between urban and rural areas in terms of economic development, infrastructure, environmental deviation, and social relations. These gaps not only directly affect wellbeing but also may have a moderating or interactive impact on religious behavior and psychological effects. However, the current literature has not reached a consistent conclusion on whether the differences between urban and rural areas have positive, negative or ineffective effects. Compared with rural residents, urban residents may be less involved in religious activities because of their busy work, or they may be trapped by work pressure and need to seek more religious help. These contradictions require research for clarification and explanation.

Based on existing research, this study discusses the relationship between religious belief, urban–rural areas, and subjective wellbeing. Accordingly, we propose the following research hypotheses.

**Hypothesis 4:** The association between the type of religious belief and the frequency of religious activity may be influenced by urban–rural areas.
**Hypothesis 5:** The association between the frequency of religious activities and subjective wellbeing may be influenced by urban–rural areas.

## Materials and methods

### Data sources

This research adopted a quantitative research method with data obtained from the TSCS. The TSCS is a sample survey research program conducted throughout Taiwan. It is administered every 5 years, with two surveys per year. This study used survey data from the fourth “religion” survey of the seventh phase of this program in 2018 for statistical analysis. The survey sample included people with household registration in Taiwan who were older than 18 years. By adopting “stratified multistage probability proportional to size sampling,” 4,096 samples were selected, and 1,842 valid samples were finally included. Among the 1,842 valid respondents, only 13.25% claimed to have no religious belief, while diffused religion accounted for three-quarters (74.97%) of the respondents who declared that they had religious beliefs.

### Measurements

#### Subjective wellbeing

The measurement of subjective wellbeing has been very well established, such as the BIT/CIT by Diener (Su et al., 2014) or the SHS by Lyubomirsky (Lyubomirsky and Lepper, 1999). Moreover, there are scales designed specifically for Chinese people, such as CHI (Lu, 2010) and SWBS-cc (Huang and Xing, 2019). These measures use scale design and cover the concepts of optimism, self-efficacy, support, community, and meaning. The TSCS 2018 Religion Questionnaire was used in this study. Although the questionnaire does not directly refer to the classical scale questions to measure subjective wellbeing, question 95 provides a complete set of scales (the four questions are as follows: I can adapt no matter how life changes; Whatever happens, I will be there to look on the positive side; I can recover easily from illness, injury, or other shocks; and I know where to find help in times of crisis), which are similar to the related topics of optimism, self-efficacy, and support in the classical scale. In this study, the four subtopics were used to generate a subjective wellbeing perception factor variable by principal component analysis. The characteristic root of this factor is 2.38, the variance contribution rate is 59.47%, and the rotated factor loadings are more than 0.68. Moreover, we computed the interitem correlations of these four items, and Cronbach's alpha was 0.7660, which indicates strong reliability. Therefore, we used this as a proxy measure for subjective wellbeing.

#### Types of religious belief and frequency of religious activities

Based on the diverse characteristics of religious beliefs in Taiwan, this study referred to Yang Qingkun's research on religious types in China (Yang, 1967) and divided religions into diffused religions and institutional religions. In this study, respondents in the “folk faith” category of the survey were considered to follow diffused religion, and all other specific religions or worship objects that could be identified were classified as institutional religion. The frequency of religious activities was coded as an ordinal variable using question number 32 of the questionnaire (1 = “almost nothing”; 8 = “several times a week”).

#### Urban–rural areas

From 2005 to 2015, the TSCS used eight variables, namely “employed population percentage in agriculture, forestry, fishery, and animal husbandry;” “employed population percentage in the industry;” “occupation level: percentage of the professional and supervisory population;” “percentage of the population aged 15–64;” “percentage of the population aged 65 and above;” “percentage of the population with college and above education;” “population density;” and “population growth in 5 years,” to divide 358 townships and urban areas in Taiwan into seven clusters to distinguish urban–rural areas in different administrative regions of Taiwan.

#### Control variables

Commonly used demographic variables were used as control variables in this study, including gender, age, and marital status. Gender still plays an important role in subjective wellbeing studies (Batz and Tay, 2018), even if findings remain inconsistent. Age affects subjective wellbeing due to social network sizes and life course stages (Bruine de Bruin et al., 2020). Married people have better wellbeing than those who are single, separated, divorced, or widowed (Wadsworth, 2016).

In addition, differences in socioeconomic status are often used to assess the impact on subjective wellbeing. A meta-analysis noted that the subjective socioeconomic status (named “self-class”) and subjective wellbeing were positively associated (Tan et al., 2020), and education attainment will also exist as an objective measure of socioeconomic status as a control variable in these studies (Zell et al., 2018). Employment status is also used in the discussion because of its stability and security changes (Bonanomi and Rosina, 2020). The variables mentioned before are all controlled in this study.

### Analytic strategy

The data were analyzed using the bruceR package in R, which includes the “PROCESS()” function and is based on the SPSS PROCESS macro developed by Hayes (2018), in conjunction with other R packages for multilevel mediation and moderation effects. In the model estimation process, 2,000 simulations were performed using the Markov chain Monte Carlo (quasi-Bayesian) method and evaluated using 95% confidence intervals.

## Results

### Descriptive statistics

Descriptive statistics of the variables involved in this study are presented in Table 1. Because most of the variables used in this study belong to categorical variables, correlation analysis among variables was not performed. Tables 1, 2 show that the average age of the respondents was 49.92 years, the percentage of women was 51.88, the average years of education was 12.51 years, and respondents living in urban cores and industrial or commercial areas accounted for 47.31%.

**Table 1:** Descriptive statistics of quantitative variables.

**Quantitative variable**	**Observed value**	**Mean value**	**Standard deviation**	**Min**	**Max**
Wellbeing	1,557	0.03	0.99	−3.21	1.48
ReliActFreq	1,595	2.97	1.99	1	8
Age	1,598	49.92	17.17	19	101
EducationYear	1,537	12.51	4.00	1	26
SelfClass	1,555	5.19	1.60	1	10

**Table 2:** Descriptive statistics of qualitative variables.

**Qualitative variable**	**Observed value**	**Sort**	**Percent**	**Sort**	**Percent**
ReligionType	1,598	1/Diffuse	74.97%	2/Institutional	25.03%
RegionType	1,598	1/Urban	47.31%	2/Rural	52.69%
Gender	1,598	1/Male	48.12%	2/Female	51.88%
Marriage	1,598	1/Married	56.82%	2/Others	43.18%
Job	1,598	0/NotFull	44.18%	1/Fulltime	55.82%

### Measurements

**Figure 1:**
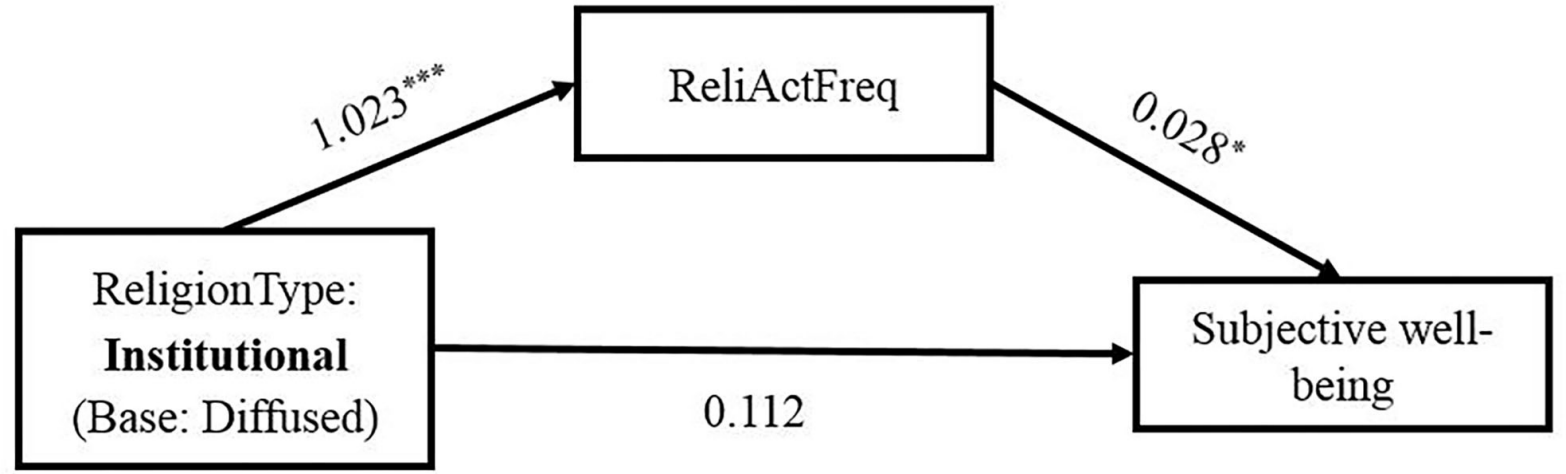
The mediation model. ReliActFreq, religious activity frequency. *p < 0.05, **p < 0.01, ***p < 0.001.

This study analyzed the influence of the type of religious belief on subjective wellbeing using the bruceR package and examined the mediating effect of religious activity frequency. Table 3 displays the regression model results of the mediation effect, and Table 4 presents the test results of the mediation effect. Compared with diffused religion, institutional religion significantly increased the frequency of respondents' participation in religious activities (β = 1.023, *p* < 0.001, CI = 0.031~0.236), and the frequency of religious activity indirectly improved subjective wellbeing (β = 0.028, *p* < 0.08, CI = 0.003~0.054). However, since institutional religion does not directly have a notable impact on subjective wellbeing compared with diffused religion, the frequency of religious activities had a fully mediating effect between the type of religious belief and subjective wellbeing (indirect effect = 0.029, *p* < 0.05, CI = 0.004~0.056; gross effect = 0.133, *p* < 0.05, CI = 0.031~0.236). Research hypotheses 1–3 were verified. Figure 1 shows the schematic presentation of the mediation model.

**Table 3:** Regression models of mediating effect.

	**(1) Wellbeing**	**(2) ReliActFreq**	**(3) Wellbeing**
ReligionType: Institutional	0.131*	1.023***	0.102
	(0.053)	(0.107)	(0.054)
ReliActFreq			0.028*
			(0.013)
RegionType: Rural	0.175**	0.027	0.173**
	(0.061)	(0.125)	(0.061)
Age	0.008***	0.013**	0.008***
	(0.002)	(0.004)	(0.002)
Gender: Female	−0.094	0.099	−0.096
	(0.050)	(0.102)	(0.050)
Marriage: Married	−0.106	0.155	−0.110*
	(0.054)	(0.110)	(0.054)
Job: FullTime	0.160**	−0.062	0.161**
	(0.056)	(0.113)	(0.056)
EduYear	0.009	0.000	0.009
	(0.008)	(0.017)	(0.008)
SelfClass	0.125***	0.074*	0.123***
	(0.017)	(0.034)	(0.017)
(Intercept)	−0.012	2.891***	−0.008
	(0.088)	(0.226)	(0.089)
Marginal *R*^2^	0.078	0.079	0.080
Conditional *R*^2^	0.096	0.124	0.100
AIC	4,106.591	6,179.907	4,110.605
BIC	4,164.874	6,238.190	4,174.186
Num. obs.	1,478	1,478	1,478
Num. groups: Region	6	6	6
Var: Region (Intercept)	0.018	0.190	0.019
Var: Residual	0.897	3.671	0.895

1. Baselines for the qualitative variables are as follows: Diffused(ReligionType), Urban(RegionType), NotMarried(Marriage), NotFull (Job) and Male(Gender).

2. Unstandardized regression coefficients are displayed, with standard errors within parentheses. *p < 0.05. **p < 0.01. ***p < 0.001.

**Table 4:** Effects of mediation model.

	**Effect**	**S.E**.	**[MCMC 95% CI]**
Indirect(ab)	0.029*	−0.013	[0.004, 0.056]
Direct(c')	0.104	−0.054	[−0.003, 0.210]
Total(c)	0.133*	−0.053	[0.031, 0.236]

*p < 0.05.

### Moderated mediating model analysis

The systematic discrepancy between urban and rural areas may moderate the relationship between religious belief and subjective wellbeing, so the moderating effect of urban–rural areas needs to be tested by the bruceR package.

**Figure 2:**
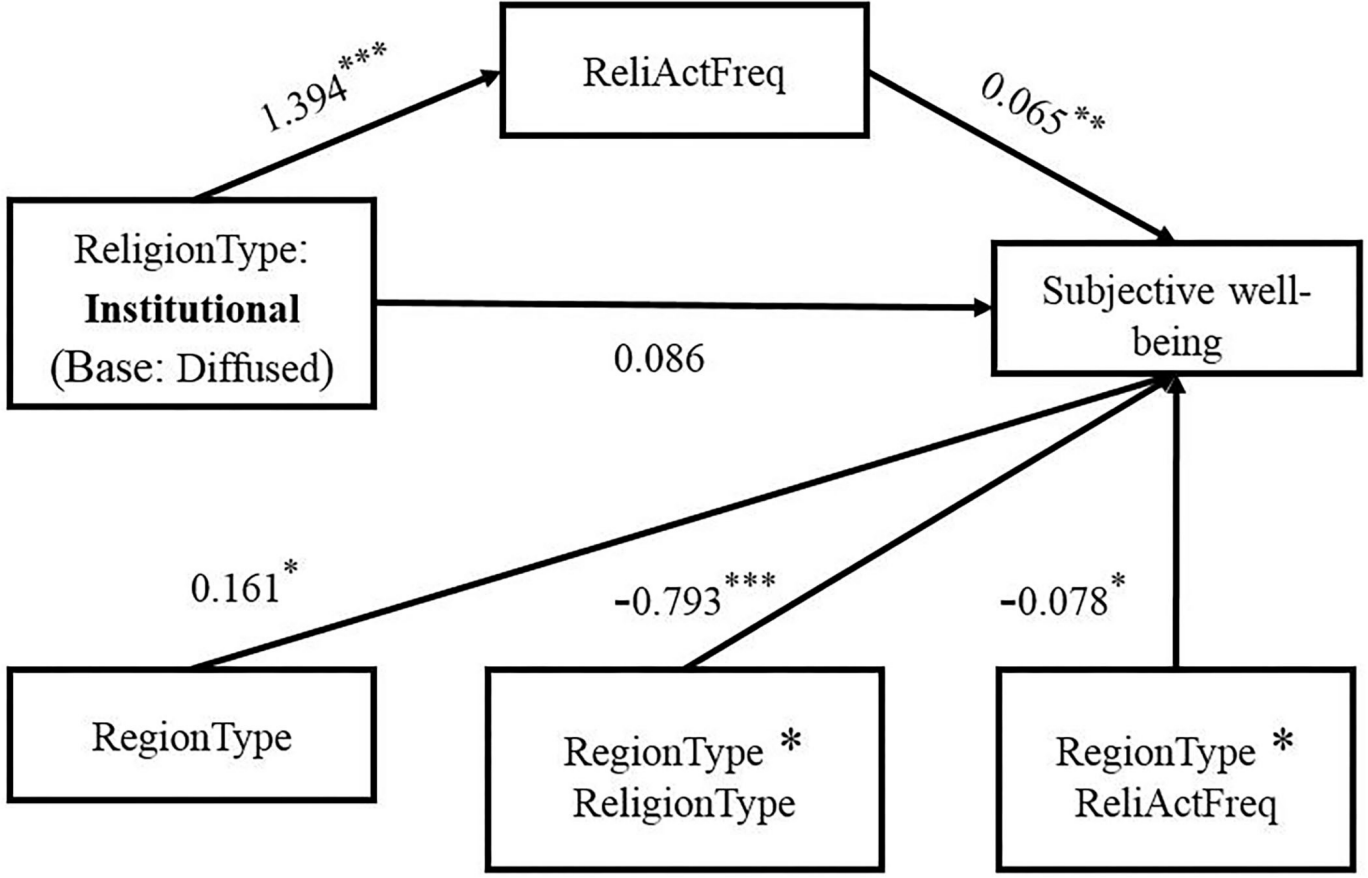
The moderated mediation model. ReliActFreq, religious activity frequency. *p < 0.05, **p < 0.01, ***p < 0.001.

The results of the mediation model (Figure 2) with moderating variables reveal that with regard to the relationship between urban–rural areas and subjective wellbeing, religious believers in rural areas had significantly higher wellbeing than those in urban areas (β = 0.161, *p* < 0.05, CI = 0.042~0.281). With further inclusion of the frequency of religious activity as a mediating variable, the association between types of religious belief and frequency of religious activity participation was significant (β = 1.394, *p* < 0.001, CI = 1.108 to 1.680), and this path was moderated by the urban–rural area variable (β = −0.793, *p* < 0.001, CI = −1.205 to −0.382) (Table 5). Furthermore, the frequency of participation in religious activity had a positive impact on subjective wellbeing (β = 0.065, *p* < 0.01, CI = 0.031~0.098), and urban–rural areas had a moderating effect on this path (β = −0.078, *p* < 0.01, CI = −0.127~ −0.029).

**Table 5:** Regression models of moderated mediating effect.

	**(4) Wellbeing**	**(5) ReliActFreq**	**(6) Wellbeing**
ReligionType: institutional	0.117*	1.394***	0.086
	(0.053)	(0.146)	(0.054)
RegionType: rural		0.064	0.161**
		(0.125)	(0.061)
ReligionType: institutional		−0.793***	
* RegionType: rural		(0.210)	
ReliActFreq			0.065***
			(0.017)
ReliActFreq * RegionType: rural			−0.078**
			(0.025)
Age	0.008***	0.012**	0.008***
	(0.002)	(0.004)	(0.002)
Gender: female	−0.101*	0.086	−0.102*
	(0.050)	(0.102)	(0.050)
Marriage: married	−0.101	0.141	−0.111*
	(0.055)	(0.110)	(0.054)
Job: FullTime	0.169**	−0.062	0.165**
	(0.056)	(0.112)	(0.056)
EducationYear	0.005	0.003	0.007
	(0.008)	(0.017)	(0.008)
SelfClass	0.123***	0.074*	0.125***
	(0.017)	(0.034)	(0.017)
(Intercept)	0.091	2.855***	−0.001
	(0.085)	(0.211)	(0.087)
Marginal *R*^2^	0.072	0.085	0.086
Conditional *R*^2^	0.095	0.121	0.104
AIC	4,108.924	6,169.040	4,108.541
BIC	4,161.908	6,232.621	4,177.421
Num. obs.	1,478	1,478	1,478
Num. groups: region	6	6	6
Var: region (Intercept)	0.023	0.150	0.018
Var: residual	0.901	3.641	0.890

1. Baselines for the qualitative variables are as follows: Diffused(ReligionType), Urban(RegionType), NotMarried(Marriage), NotFull (Job) and Male(Gender).

2.Unstandardized regression coefficients are displayed, with standard errors within parentheses. *p < 0.05. **p < 0.01. ***p < 0.001.

Table 6 presents the test of the CIE. Whether in urban or rural areas, respondents in institutional religions participated in religious activities more frequently than those with diffused religion, and believers in urban areas (β = 1.394, *p* < 0.001, CI = 1.108~1.680) showed a stronger positive effect than those in rural areas (β = 0.6, *p* < 0.001, CI = 0.299~0.901). In addition, the urban respondents had a stronger effect of religious activity frequency on promoting wellbeing (β = 0.065, *p* < 0.01, CI = 0.031~0.098), while the rural respondents did not significantly differ from the average. In the mediation model with moderating variables, urban respondents also had a positive moderating effect on full mediation (β = 0.009, *p* < 0.01, CI = 0.040~0.143), which was not the case for rural respondents.

**Table 6:** Main effects of moderated mediation model.

				**Effect**	**S.E**.	**[95% CI]**
Mediating effect	X → Y	Direct (c')		0.086	−0.054	[−0.019, 0.193]
Moderating effect	X → M	Conditional (a)	Urban	1.394***	0.146	[1.108, 1.680]
			Rural	0.6***	0.154	[0.299, 0.901]
	M → Y	Conditional (b)	Urban	0.065***	0.017	[0.031, 0.098]
			Rural	−0.013	0.019	[−0.050, 0.023]
	X → M → Y	Indirect (ab)	Urban	0.09***	0.026	[0.040, 0.143]
			Rural	−0.008	0.011	[-0.033, 0.013]

***p < 0.001.

This study further conducted an *F*-test for determining the interaction effects of the moderating and independent variables separately with the mediating variables (Table 7), which showed that both variables were significant. Thus, research hypotheses 4 and 5 are verified.

**Table 7:** Interaction effects of moderated mediation model.

**Pathway**	**Interaction effect**	***F***	***df1***	***df2***
X → M	ReligionType * RegionType	14.33***	1	1,461
M → Y	ReliActFreq * RegionType	9.63***	1	1,467

***p < 0.001.

## Discussion

The core goal of this study is to analyze the effects of different types of religion on subjective wellbeing and its intrinsic influence mechanism. The correlation analysis shows that there is no significant correlation between the type of religion and subjective wellbeing; however, people who believe in institutional religion are more inclined to participate in religious activities than individuals who believe in diffused religion, which has a prominent positive correlation with wellbeing. Hence, the frequency of participation in religious activities plays a complete mediating role between the type of belief and subjective wellbeing. In addition, the urban–rural type plays a moderating role between the type of religion and subjective wellbeing.

### Analysis of the mediating effect of religious activity frequency between the type of religious belief and subjective wellbeing

Based on the test results, belief in institutional religion did not make respondents happier than belief in diffused religion. In other words, no statistically direct causality existed between the type of religion of the respondents and their wellbeing. This conclusion is consistent with some informed research, that is, different religions have no impact on individuals' wellbeing. Further analysis showed that the frequency of believers' participation in religious activities is an important mediating variable, revealing an indirect correlation between the type of religious belief and subjective wellbeing. This means that different types of religious belief cause distinctions in the frequency of participation in religious activities, which, in turn, lead to changes in wellbeing. Since respondents who believe in institutional religion have a higher frequency of participating in religious activities, this has an eminent positive effect on their wellbeing.

In response to these data results, we presume that different types of religions have great disparities in the pattern and frequency of organizing religious activities. For institutional religion, the time for religious activities is relatively fixed. For example, Islam and Christianity hold religious activities at a fixed time. Furthermore, the religious rituals of institutional religion are also fixed. Institutional religion is more normative and stable in the organization and management of religious activities, and this regular participation in religious activities in fixed places may make believers feel a sense of belonging and satisfaction and provide psychological comfort. As noted in previous studies, believers have different degrees of religious commitment to different religions, that is, the degree of participation and devotion to religion varies. From the perspective of different levels of believers' commitment, on the one hand, fixed places and stable religious rituals can attract believers for a longer period of time and provide them with a steady religious experience; on the other hand, the high frequency of religious activities indicates that believers are willing to invest more resources in the religion they believe in. With the continuous dedication and investment of time, energy, and money, believers' sense of dependence on religion will also be strengthened, which may lead to transformations in their wellbeing.

### Analysis of the moderating effect of urban–rural areas

This study also examined the moderating effect of urban–rural areas on the relationship between types of religious beliefs and subjective wellbeing. We found that urban–rural areas have a moderating effect on the relationship between the types of religious beliefs and the frequency of religious activities as well as the relationship between the types of religious beliefs and subjective wellbeing.

On the one hand, urban–rural areas affect the frequency of religious activities in different religions. In line with existing qualitative research findings, respondents living in urban cores and industrial or commercial areas are more inclined to participate in religious activities of institutional religions. Urban residents generally face multiple pressures, including housing, transportation, education, and medical care, so they have a more prominent demand for ways to adjust to psychological pressure. Institutional religions are more likely to be favored by urban respondents due to the normative nature of their rituals and places. Conversely, the rhythm of rural life is relatively slow, especially because some rural residents are still in a state of meeting survival demands. Moreover, rural areas have a historical tradition of embracing folk beliefs. Even if rural residents claim to believe in institutional religion, they will not feel extra satisfaction because of changes in the frequency of religious activities. Therefore, urban–rural areas play a moderating role in the relationship between types of religious beliefs, the frequency of religious activities, and subjective wellbeing.

On the other hand, in the process by which the frequency of participation in religious activities influences subjective wellbeing, the differences between urban and rural areas are mainly reflected in the formation and changes of social networks. More precisely, respondents living in the urban core and industrial or commercial areas participate in religious activities more frequently, which is positively related to wellbeing. In light of this result, we suggest that the multifrequency religious activities in urban areas are more likely to establish new social networks; therefore, people can expand their social circle and obtain satisfaction from it. As noted by existing studies, some religious doctrines describe the relationship between believers as brothers and sisters, which provides individuals with a psychological sense of dependence and belonging in urban areas where interpersonal relations are relatively cold. By contrast, rural population mobility is relatively weak, and social relations are fixed. Therefore, frequent religious activities will not necessarily bring remarkable changes to social networks; therefore, personal psychological benefits and satisfaction are unlikely to be produced.

### Contributions and limitations

The sample selected in this study involved survey data from Taiwanese residents. Taiwan has relatively specific religious traditions and folk culture. Primitive religious beliefs prevail among the aboriginal people in Taiwan. This folk religion is mainly in the form of nature worship, totem worship, and ancestor worship and differs greatly from institutional religions with standardized doctrines and religious rituals. Therefore, the research results of this study have value in explaining Asian regions with diversified religious beliefs. In addition, this study reveals the mechanism of the relationship between the type of religious belief and subjective wellbeing.

This study still has several limitations. First, there is a gap between the measurement of subjective wellbeing and the classical scale. This study adds several variables related to subjective wellbeing as control variables but cannot provide an accurate measurement. Second, the data used in this study are cross-sectional data. If a follow-up survey can be conducted on a relevant topic, the causal effect can be further explored to improve the explanatory power and scalability of the empirical results. Third, the impact of specific religious differences on subjective wellbeing needs to be further explored. Different religions differ greatly in their doctrines and rituals, and this heterogeneity can help academics reveal the psychological mechanisms of religion at the empirical level.

## Conclusion

The rapid development of the economy and technology has completely changed people's lifestyles, effectively responding to the increasingly diversified needs of individuals and, in general, improving the material quality of individuals' lives. However, existing studies note that the progress of material life is not the only source of subjective wellbeing and spiritual comfort, pleasure, and satisfaction are equally critical to improve subjective wellbeing. As a special ideology, religion responds to the internal needs of believers in different ways and provides spiritual comfort for different people with the help of belief systems and organizational rituals.

This study tested a mediation model to examine the relationship between the type of religious belief and subjective wellbeing. The results show that in the case of Chinese Taiwan, no significant correlation exists between the type of religious belief and subjective wellbeing. However, the frequency of religious activities has a complete mediating effect based on further research that reveals the internal mechanism by which it affects wellbeing. These findings can help us to better understand the internal mechanism and logical relationship of the mental impact on subjective wellbeing.

## Data availability statement

The original contributions presented in the study are included in the article/supplementary material, further inquiries can be directed to the corresponding authors.

## Ethics statement

Written informed consent was obtained from the individual(s) for the publication of any potentially identifiable images or data included in this article.

## Author contributions

YD: conceptualization and original draft. WH: investigation and methodology. YJ: translation and editing. All authors read and approved the final manuscript.

## References

[B1] Abdel-Khalek A. M. (2006). Happiness, health, and religiosity: significant relations. Mental Health Relig. Cult. 9, 85–97. 10.1080/1369467050004062524327187

[B2] Abdel-Khalek A. M. Naceur F. (2007). Religiosity and its association with positive and negative emotions among college students from Algeria. Mental Health Relig. Cult. 10, 159–170. 10.1080/13694670500497197

[B3] Abu-Raiya H. Agbaria Q. (2016). Religiousness and subjective well-being among israeli-palestinian college students: direct or mediated links? Soc. Indic. Res. 126, 829–844. 10.1007/s11205-015-0913-x

[B4] Bänziger S. Janssen F. Hutsebaut D. Dezutter J. (2005). Religion and mental health: aspects of the relation between religious measures and positive and negative mental health. Arch. Psychol. Relig. 27, 19–44. 10.1163/008467206774355402

[B5] Batz C. Tay L. (2018). “Gender differences in subjective wellbeing,” in Handbook of wellbeing, eds E. Diener, S. Oishi, and L. Tay (Salt Lake City, UT: DEF Publishers).32326600

[B6] Blanchflower D. G. Oswald A. J. (2004). Money, sex and happiness: an empirical study. Scand. J. Econ. 106, 393–415. 10.1111/j.0347-0520.2004.00369.x

[B7] Bonanomi A. Rosina A. (2020). Employment status and wellbeing: a longitudinal study on young italian people. Soc. Indicat. Res. 161, 1–18. 10.1007/s11205-020-02376-x

[B8] Bruine de Bruin W. Parker A. M. Strough J. (2020). Age differences in reported social networks and wellbeing. Psychol. Aging 35, 159. 10.1037/pag00000415 31697096 PMC7122684

[B9] Clark A. E. Oswald A. J. (1994). Unhappiness and unemployment. Econ. J. 104, 648–659.

[B10] Deaton A. (2008). Income, health, and wellbeing around the world: evidence from the Gallup World Poll. J. Econ. Perspect. 22, 53–72. 10.1257/jep.22.2.53 19436768 PMC2680297

[B11] Di Tella R. MacCulloch R. J. Oswald A. J. (2001). Preferences over inflation and unemployment: evidence from surveys of happiness. Am. Econ. Rev. 91, 335–341. 10.1257/aer.91.1.335

[B12] Dolan P. (2014). Happiness by Design: Change What You Do, Not How You Think. London: Penguin Books.

[B13] Dolan P. Peasgood T. White M. (2008). Do we really know what makes us happy? A review of the economic literature on the factors associated with subjective wellbeing. J. Econ. Psychol. 29, 94–122. 10.1016/j.joep.2007.09.001

[B14] Easterlin R. A. (1974). Does Economic Growth Improve the Human Lot? Some Empirical Evidence. Nations and Households in Economic Growth. Cambridge, MA: Academic Press.

[B15] Easterlin R. A. Angelescu L. (2011). Modern economic growth and quality of life: cross sectional and time series evidence. Handbook Soc. Indicat. Qual. Life Res. 10, 113–136. 10.1007/978-94-007-2421-1_6

[B16] Gasper D. (2010). Understanding the diversity of conceptions of wellbeing and quality of life. J. Soc. Econ. 39, 351–360. 10.1016/j.socec.2009.11.006

[B17] Hayes A. F. (2018). Partial, conditional, and moderated moderated mediation: quantification, inference, and interpretation. Commun. Monogr. 85,4–40. 10.1080/03637751.2017.1352100

[B18] Helliwell J. F. (2003). How's life? Combining individual and national variables to explain subjective wellbeing. Econ. Model. 20,331–360. 10.1016/S0264-9993(02)00057-3

[B19] Hoogeveen S Sarafoglou A van Elk M. Wagenmakers E. J. (2022). A many-analysts approach to the relation between religiosity and well-being. Religion Brain Behav. 1–47. 10.1080/2153599X.2022.2070255

[B20] Huang L. Xing Z. (2019). A nationwide study on subjective wellbeing scale for Chinese citizens (SWBS-cc). Int. J. Appl. 9, 117–127. 10.5923/j.ijap.20190905

[B21] Hudson J. (2006). Institutional trust and subjective wellbeing across the EU. Kyklos 59, 43–62. 10.1111/j.1467-6435.2006.00319.x

[B22] James W. Chris H. (2013). Religion, deprivation and subjective wellbeing: testing a religious buffering hypothesis. *Int. J. wellbeing* 3. Available online at: https://internationaljournalofwellbeing.org/index.php/ijow/article/view/207

[B23] Katz P. R. (2003). Religion and the state in post-war Taiwan. China Q. 174, 395–412. 10.1017/S000944390300024X

[B24] Knight S. G. (2009). Subjective wellbeing and its determinants in rural China. China Econ. Rev. 20, 635–649. 10.1016/j.chieco.2008.09.00327349854

[B25] Laliberte A. (2016). Managing religious diversity in China: contradictions of imperial and foreign legacies. Stud. Relig. Sci. Relig. 45, 495–519. 10.1177/0008429816659351

[B26] Levinson A. (2012). Valuing public goods using happiness data: the case of air quality. J. Public Econ. 96,869–880. 10.1016/j.jpubeco.2012.06.007

[B27] Lewis C. A. Cruise S. M. (2006). Religion and happiness: consensus, contradictions, comments and concerns. Mental Health Relig. Cult. 9,213–225. 10.1080/13694670600615276

[B28] Liu M. B. (2008). On the characteristic of coexisting of folk belief and institutional religion: taking the sacrificial folk belief of two villages as examples. J. South-Central Univ. Natl. 4, 1–15. 10.19898/j.cnki.42-1704/c.2008.02.004

[B29] Lu L. (2010). Who is happy in Taiwan? The demographic classifications of the happy person. Psychologia 53, 55–67. 10.2117/psysoc.2010.55

[B30] Lu Y. Johnson B. Stark R. (2008). Deregulation and the religious market in Taiwan: a research note. Sociol. Q. 49, 139–153. 10.1111/j.1533-8525.2007.00109.x

[B31] Lyubomirsky S. Lepper H. S. (1999). A measure of subjective happiness: preliminary reliability and construct validation. Soc. Indicat. Res. 46, 137–155.

[B32] Oishi S. Diener Ed. Lucas R. E. (2021). Subjective Well-Being: The Science of Happiness and Life Satisfaction. The Oxford Handbook of Positive Psychology, 3rd edn., eds C. R. Snyder, S. J. Lopez, L. M. Edwards, S. C. Marques (Oxford Academic), 254–264. 10.1093/oxfordhb/9780199396511.013.14

[B33] Peterson C. Ruch W. Beermann U. Park N. Seligman M. E. (2007). Strengths of character, orientations to happiness, and life satisfaction. J. Posit. Psychol. 2,149–156. 10.1080/17439760701228938

[B34] Qi Y. (2019). The political involvement of religious organizations from authoritarian to democratic consolidation periods: a case study of Taiwan and South Korea. Chin. Polit. Sci. Rev. 67, 51–86. 10.6229/CPSR.201906_67.0002

[B35] Reid D. Tamaru N. (1996). Religion in Japanese Culture: When Living Traditions Meet a Changing World. Kodansha USA Inc Press.

[B36] Stark R. Finke R. (2000). Acts of Faith: Explaining the Human Side of Religion. California: Univ of California Press.

[B37] Su R. Tay L. Diener E. (2014). The development and validation of the comprehensive inventory of thriving (CIT) and the brief inventory of thriving (BIT). Appl. Psychol. Health wellbeing 6, 251–279. 10.1111/aphw.12027 24919454

[B38] Tan J. J. Kraus M. W. Carpenter N. C. Adler N. E. (2020). The association between objective and subjective socioeconomic status and subjective wellbeing: a meta-analytic review. Psychol. Bull. 146, 970. 10.1037/bul0000258 33090862

[B39] Wadsworth T. (2016). Marriage and subjective wellbeing: how and why context matters. Soc. Indicat. Res. 126, 1025–1048. 10.1007/s11205-015-0930-9

[B40] Weber M. (2002). The Protestant Ethic and the Spirit of Capitalism. London: Penguin.

[B41] Wei Z. (2012). Economic inequality, status perceptions and subjective wellbeing in China's transitional economy. Res. Soc. Stratif. Mobil. 30, 433–450. 10.1016/j.rssm.2012.07.001

[B42] Yang C. K. (1967). Religion in Chinese Society: A Study of Contemporary Social Functions of Religion and Some of their Historical Factors. California: Univ of California Press.

[B43] Yang F. (2006). The red, black, and gray markets of religion in China. Sociol. Q. 47, 93–122. 10.1111/j.1533-8525.2006.00039.x

[B44] Zell E. Strickhouser J. E. Krizan Z. (2018). Subjective social status and health: a meta-analysis of community and society ladders. Health Psychol. 37, 979. 10.1037/hea0000667 30234357

